# Genetic association of *IL-1B-511* (C/T), *TNF-A-308* (G/A) and *IL-10-1082* (G/A) with gastric cancer risk in Colombian populations

**DOI:** 10.3332/ecancer.2026.2107

**Published:** 2026-04-07

**Authors:** Kevin Guzmán, Vanessa Pabón, Andrés Angulo, Luis E Bravo, Arsenio Hidalgo, Carol Rosero, Alvaro Pazos

**Affiliations:** 1 Grupo Salud Publica, Centro de Estudios en Salud, Universidad de Nariño, Pasto 520001, Colombia; 2 Registro Poblacional de Cáncer de Cali, Department of Pathology, School of Medicine, Universidad del Valle, Cali 760043, Colombia; 3 Departamento de Matematicas y Estadistica, Universidad de Nariño, Pasto 520001, Colombia; 4 Facultad de Medicina, Universidad Cooperativa de Colombia, Pasto 520001, Colombia; 5 Departamento de Biología, Universidad de Nariño, Pasto 520001, Colombia

**Keywords:** gastric cancer biomarkers, interleukin 1β, interleukin 10, gastric cancer, gastric atrophy, Helicobacter pylori

## Abstract

**Background:**

Gastric cancer (GC) incidence varies markedly across Colombia. Host genetic polymorphisms in cytokine genes modulate the inflammatory response to Helicobacter pylori and may influence disease progression.

**Objective:**

To evaluate IL-1B-511, IL-10-1082 and TNF-A-308 polymorphisms and their association with gastric precursor lesions in Colombian populations with contrasting GC risk.

**Methods:**

We studied 160 dyspeptic patients from Túquerres (high risk) and 105 from Tumaco (low risk). Gastric lesions were classified using the Sydney system. Cytokine polymorphisms were genotyped by polymerase chain reaction–restriction fragment length polymorphism and sequencing. Associations were assessed using logistic regression.

**Results:**

The IL-1B-511TT and IL-10-1082AA genotypes were significantly more frequent in Túquerres than in Tumaco (p < 0.05). Individuals carrying the IL-10-1082AG genotype had a significantly higher risk of gastric atrophy (odds ratio = 5.45; 95% confidence interval, 1.0–30.59).

**Conclusion:**

IL-10-1082AG is associated with increased risk of gastric atrophy in high-risk Colombian populations, supporting its potential role as a biomarker of GC susceptibility.

## Background

Gastric cancer (GC) is the fifth most frequent malignant neoplasia and the third leading cause of death by cancer worldwide. Also at a global scale, the incidence and mortality rates and the GC risk vary [1, 2]. In Colombia, GC represents the main cause of death by cancer and presents a marked geographical pattern with respect to its incidence distribution, being higher in mountainous regions and lower in coastal areas of the national territory [3, 4].

*Helicobarter pylori* (*H. pylori*) infection is the principal etiological factor involved in the development of GC [2, 5–7]; however, the high infection prevalence is not a predictor for the disease incidence [1, 6, 8, 9]. There are other risk factors that are involved with the GC pathogenesis, such as the environmental and hygiene-sanitary factors, diet, host-*H. pylori* coevolution and human genetic susceptibility [8–13].

The GC human genetic susceptibility is mediated by pro-inflammatory and anti-inflammatory cytokine polymorphisms that participate in the immune response to *H. pylori* infection. The expression persistence of pro-inflammatory interleukin (IL) polymorphisms is an important factor that contributes to the transformation from a normal gastric mucosa to one displaying precancerous lesions [14–17]. Nevertheless, the evidence that links these polymorphisms with gastric lesions is dependent on the analysed population. For instance, some studies demonstrated a positive association between the genotypes *IL-1B-511* and GC risk in Caucasian populations [15, 18, 19], whereas no association of this genotype was found in Asian [20, 21] and Latin American populations from Costa Rica [22], Brazil [23] and Venezuela [24], in contrast to what was reported for some populations in Colombia where an association was found [25, 26]. The genetic susceptibility due to polymorphisms in pro-inflammatory cytokine genes *IL-1B-511, FNT-A-308* and anti-inflammatory cytokines *IL-1RN* and* IL-10* are considered an essential factor in the malignant transformation of the normal gastric mucosa. These cytokines participate in the immune response to infections and modulate the type of severity of the gastric inflammation [14, 18]. The opposite results in the analysed associations might indicate that the ethnic differences in the populations influence the distribution of the allelic and genotypic frequencies of these polymorphisms [19, 26, 27].

The Department of Nariño in Colombia represents an important study model to elucidate the GC etiopathogenesis due to its unique geographical, sociocultural and ethnic situation, with an Andean zone of high mountains inhabited by a population of Amerindian origin (67%) and a high GC incidence, which is considered one of the highest in the world (150/100.000 inhabitants). Nariño also has a coastal zone with a population of mostly African origin (58%) with a low GC incidence (6/100.000 inhabitants) [3, 9]. Taking into account the contrasting GC risk in these populations and the contrasting association of cytokine polymorphisms, we investigate what is the relationship of *IL-1B-511*, *IL10-1082* and *TNF-A-308* polymorphisms with gastric lesions in two Colombian populations with opposite risk of GC.

## Methods

### Subjects and samples

We included 265 subjects between the ages of 19–67, with dyspepsia symptoms. Of these, 44.2% (63/160) of men were from Túquerres, a population located in the Andean mountains of Colombia with a high prevalence of *H. pylori* and preneoplastic lesions. The remaining 41% (38/105) were men from Tumaco, a population located on the Pacific coast with a low GC risk [9]. We analysed 52 patients with GC precursor lesions in these two populations: eight with atrophic chronic gastritis (ACG) and 44 with intestinal metaplasia (IM) and 213 control group patients with non-atrophic gastritis (NAG). We obtained four biopsies from the gastric antrum and gastric body for their histopathological evaluation and *H. pylori* diagnosis. We obtained 5 mL of total blood for the evaluation of biomarkers of genetic susceptibility to GC.

Posterior to the signature of the informed consent, the participants were given a survey about their sociodemographic factors. This study was approved by the Human Ethics Committee of both Universidad del Valle and Universidad de Nariño.

### Histopathology procedures

The gastric lesions were interpreted according to the Sydney classification system [28]. The presence of *H. pylori* was determined by Giemsa stain. The classified categories were: NAG, ACG and IM. Patients with NAG were defined as a control group and patients with ACG and IM as a group of cases.

### DNA extraction

Genomic DNA was extracted from blood using the Ultra Clean Blood Spin Isolation Kit (Mobio, Technologies Inc, San Diego, USA). DNA concentration was determined using NanoDropTM (2000/2000c Spectrophotometers-Thermo Scientific (Thermo Fisher Scientific, Waltham, MA, USA).

### IL-1B-511 (C/T) polymorphism

Polymerase chain reaction–restriction fragment length polymorphism (PCR-RFLPs) was used to genotype the *IL-1B-511* (C/T) polymorphism. Initially, a 304 bp fragment containing the polymorphic position 511 (C-T transition) was amplified using the primers: 5’-TGGCATTGATCTGGTTCATC-3’ and 5’-GTTTAGGAATCTTCCCACTT-3’ [29, 30], followed by digestion with AvaI restriction enzyme. The PCR conditions were the following, using a 30 µL total volume containing: 1 μL of DNA concentration between 5 and 130 ng/μL; 0.5 nM of each primer; 0.1 nM of the dNTPs mix; 0.5 mM of MgCl_2_ and 1.25 U of GoTaq DNA Polymerase (Promega^®^). Amplification conditions: 94°C/5 minutes, followed by 34 amplification cycles (94°C/0.45 second, 57°C/0.45 second and 72°C/0.45 second) and a final extension at 72°C/7 minutes, using a thermocycler (T100TM Biorad). The RFLPs of the PCR products were carried out during 16 h at 37°C using a 30.3 µL final volume containing: 10 µL PCR product, 3 U AvaI restriction enzyme (Eco881) dissolved in 2 µL 10X Tango buffer (Thermo Scientific). The digestion reaction was carried out in the presence of the normal allele (C) and generated two bands of 190 and 114 bp. The characteristic bands for each genotype were: normal homocygous individuals CC (190 and 114 bp), homozygous mutant TT (304 pb) and heterozygous CT (304, 190 and 114 bp) [29, 30]. The restriction fragments from this and the following two digestion reactions were separated by electrophoresis using 1.5% agarose gels stained with 0.4 µL GelRedTM (Biotium, 10.000X) [29].

### IL-10-1082 (G/A) polymorphism

We applied PCR-RFLPs to genotype the *IL-10-1082* (G/A) polymorphism. We first amplified a 360 bp fragment that contains the polymorphic region -1082 (G-A Transition) using the primers: 5’-CCAAGACAACACTACTAAGGCTTCTTGAGG-3’ and 5’AGGTAGTGCTCAC CATGACC-3’ [29, 30], and the obtained products were subjected to restriction reaction with BseRI. PCR was performed in a final volume of 30 µL containing 2 µL DNA at 5–130 ng/µL; 0.5 nM of each primer; 0.3 nM dNTPs mix; 0.5 mM MgCl2 and 1.5 U of GoTaq DNA Polymerase (Promega^®^). The amplification step was as follows: 95°C/10 minutes, followed by 35 cycles (95°C/0.30 second, 58°C/0.30 second y 72°C/0.45 second) and a final extension at 72°C/10 minutes using the same thermocycler as above. RFLP digestions were carried out for 45 minutes at 37°C using a 15 µL final volume, containing: 5 µL PCR product, 2 U BseRI diluted in 1.5 µL 10X NE buffer (New England, BioLabbs^®^). The digestion was produced in the presence of the normal allele (G) and the produced bands for each genotype were: GG normal homozygous individuals (320 bp), AA mutant homozygous (360 bp) and GA heterozygous (360, 320 bp) [29, 30].

### TNF-A-308 (G/A) polymorphism

PCR-RFLP was used to genotype the *TNF-A-308* (G/A) polymorphism. We initially amplified a 107 bp fragment that includes the polymorphic region -308 (G-A Transition), using the primers: 5’-AGGCAATAGGTTTTGAGGGCCAT-3’ and 5’-TCCTCCCTGCTCCGATTCCG-3’ [29, 30], followed by digestion with Nco1 restriction enzyme. The PCR reaction mix was similar to the one used for IL-1B gene. The amplification steps included: 94°C/5 minutes, 30 cycles (94°C/1 minute, 57°C/1 minute and 72°C/1 minute) and final extension at 72°C/5 minutes in the same thermocycler. The RFLPs of the PCR products were carried out for 16 h at 37°C in a final volume of 30.3 µL that contained 10 µL PCR, 3 U Nco1 enzyme diluted in 2 µL 10X Tango buffer. The digestion was developed in the presence of the normal allele (GG) that generates a 87 bp band; the heterozygous (GA) produce two bands of 87 and 107 bp, and the mutant homozygous (AA) generates a 107 bp band [29, 30].

### Statistical analysis

We evaluated the adjustment of the Hardy–Weinberg equilibrium (EH-W) using the allelic and genotypic frequencies of the control group from the two studied populations through the Markov Chain approach with 100,000 permutations in the Arlequin software version 3.5.2. We made contingency tables based on the χ^2^ test in order to determine whether there are significant differences in the genotypic and allelic distributions between the two analysed zones and to determine their relationship with the type of gastric lesion. The magnitude of the association between the genotypes and the gastric lesion was expressed as odds ratio (OR) with a 95% confidence interval (95% CI) that was estimated via binary logistic regression adjusted by sex, age, *H. pylori* infection and polymorphisms simultaneously. An association was considered significant if it showed a *p*-value ≤ 0.05. The statistical analyses were carried out using the SPSS program version 22.0.

## Results

The 52 patients with GC precursor lesions were classified as GCA [8] and IM [44], and 213 patients were placed in the control group NAG. The infection prevalence of *H. pylori* in Tumaco was 94.3%, which is significantly higher than the one in Tuquerres (85.6%) (*p* < 0.05). We observed that the type of gastric lesion is dependent on the geographical origin, with a borderline significance (*p* = 0.07). Here, the IM lesion was the more frequent in the high-risk population (68.2%) than in the low-risk population (31.8%), whereas NAG showed a higher frequency in Tumaco (75%) than in Túquerres (25%). We combined the ACG and IM cases in a single group (ACG) due to the low frequency of the number of GC precursor lesions and to carry out the statistical analysis. With respect to the sociodemographic characteristics of the patients, we found a significant difference (*p* < 0.05) between the cases and control groups for both populations in reference to sex. We observed that gastric atrophy was more frequent in male (30.7%) than in females (12.8%), and men have a three times higher risk of developing ACG compared to women, independently of their origin. A similar trend was observed with age, where the GC precursor lesions are more frequent in individuals older than 30 years of age (22.2%) compared to younger individuals (5%) (*p* = 0.01) (Table 1). In addition, individuals older than 30 years of age had a 5.4 higher risk of developing gastric atrophy compared to younger people. Finally, we did not find significant differences between *H. pylori* infection and the type of gastric lesion in individuals from the two studied populations (Table 1).

### IL1B-511, IL-10-1082 and TNF-A-308 polymorphisms

**Figure 1.**
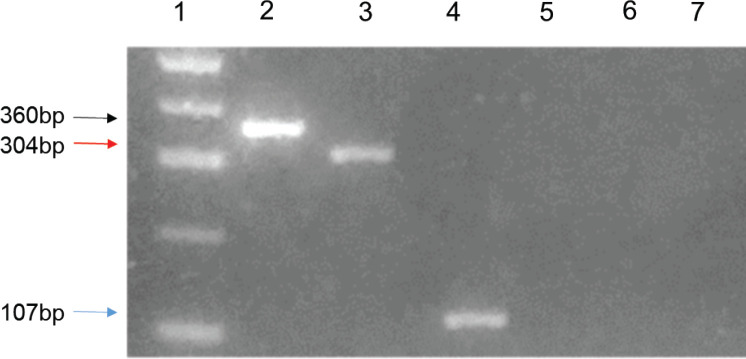
Electrophoretic pattern of the PCR products from the polymorphic regions -1082, -511 and -308 in IL10, IL1-B and TNF-A genes, respectively. 1.5% agarose gel electrophoresis. Lane 1: molecular marker (100 bp DNA ladder); lane 2: the black arrow indicates the 360 bp band that corresponds to the amplification product of -1082 region of IL-10 gene; lane 3: the red arrow indicates the 304 bp band corresponding to the amplification product or the -511 region of the IL1-B gene; lane 4: the blue arrow marks the 107 bp band that represents the amplification product of the -308 region of the gene TNF-A; lanes 5–7: negative controls for each gene.

**Figure 2.**
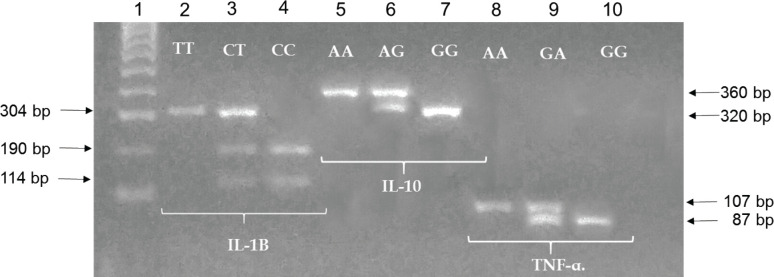
Electrophoretic pattern of the digestion products by RFLP of the polymorphic regions -511, -1082 and -308, IL1-B, IL-10 and TNF-A genes, respectively. 1.5% agarose gel electrophoresis. Lane 1: molecular marker (100 bp DNA ladder); lanes 2–4: the arrows indicate the 304, 190 and 114 bp bands of the digestion products obtained with AvaI enzyme, corresponding to the mutant homozygous genotypes (TT), heterozygous (CT) and homozygous (CC) of the -511 region of IL1-B gene. Lanes 5–7: the arrows indicate the 360 and 320 bp bands produced by digestion with BseRI restriction enzyme, which correspond to homozygous genotype (AA), heterozygous (AA) and homozygous (GG), -1082 region of IL-10 gene. Lanes 8–10: the arrows indicate the 107 and 87 bp band products of the digestion with NcoI that correspond to the AA mutant, GA heterozygous and GG homozygous normal genotypes, -308 region of the TNF-A gene.

The PCR-based amplification of the polymorphic regions of *IL-1B, IL-10* and *TNF-A* genes revealed the presence of the products with the expected polymorphic fragments according to the used primers (Figure 1). The categorisation and classification of the products from PCR-RFLPs allowed us to detect the presence of the three genotypes from each of the polymorphisms in both populations (Figure 2).

#### Frequencies of cytokine polymorphisms according to population at risk for GC

We analysed 259, 262 and 264 polymorphic profiles from *IL-1B-511, IL-10-1082* and *TNF-A-308*, respectively. It was determined that the group controls from both populations are in EH-W equilibrium for the *TNF-A-308* gene polymorphism (*p* = 0.37 for Tumaco and *p* = 1.00 for Túquerres). The *IL-10-1082* was adjusted to the EH-W equilibrium (*p* = 0.78) only in the control group from the high-risk population, which means that it is not under the influence of evolutive factors or forces that can modify it. With respect to *IL-1B-511* gene polymorphism, the EH-W was not adjusted in either of the two populations (*p* = 0.001 for Tumaco and* p* = 0.04 for Túquerres) [26].

The *IL1B-511TT* and *IL-10-1082AA* genotypes, which are carriers of the T and A risk alleles, were significantly more frequent in the GC high-risk population: Túquerres (54.4% and 63.9%) than in the low-risk population: Tumaco (36.6% and 54.3%), respectively, *p* ≤ 0.05. The distribution of the alleles showed that the patients with high GC risk had a significantly higher frequency of the *IL1B-511T* mutant alleles (72%) and *IL-10-1082A* (79%) than the frequencies found for these alleles in the low GC risk patients: Tumaco,* p* ≤ 0.05 (Table 2). We found that the *TNF-A-308AA* genotype was absent in the Túquerres population, while in the Tumaco population this genotype was only present in 2.9% of the patients. Similarly, the distribution of the A allele frequency of this genotype is almost null in the two populations (Table 2).

#### Frequencies of cytokine polymorphisms according to gastric lesion type

The distribution of the different genotypes within the control group NAG and within the study group ACG showed that the *IL1B-511, IL-10-1082* and* TNF-A-308* gene polymorphisms had no differences between the type of gastric lesion and the genotypes of (*IL*) in the two studied populations (Table 3). Independent of population, we found that the most predominant genotype in the ACG patient group was the *IL-1B-511CT* heterozygous which displayed higher frequency in Túquerres (51.6%) as well as in Tumaco (50%). In the case of the *IL-10-1082AA* genotype, carriers of the A risk allele were the most prevalent among the ACG patients in both populations, Túquerres (65.6%) and Tumaco (45%). Significant differences were not found in the total sum of frequencies of the risk genotypes versus the normal genotypes in the three studied genes and the type of gastric lesion (Table 3). It was found that the *TNF-A-308AA* genotype, carrier of the rare A allele, was absent in patients in ACG case groups from both populations, while the *TNF-A-308GG* genotype, carrier of the frequent allele, was more common in general. Since this polymorphism was absent in the case groups, it was not included in the logistic regression model (Table 3). The results of the analysis of the binary logistic regression which were adjusted simultaneously by sex, age, *H. pylori* infection and all of the polymorphisms, showed that people that inhabit the high risk zone and are carriers of the heterozygous *IL-10-1082AG* genotype presented a 5.45 higher probability of developing ACG in comparison to those who were carriers of the *IL-10-1082AA* genotype (OR = 5.4; 95% CI, 1.0–30.59). With respect to the remaining genotypes, no positive association with gastric lesion was found (Table 4).

The results of the allelic and genotypic variants evaluated by PCR-RFLP for the three cytokine genes were concordant with those found by the method of sequencing and identification of mutations. The kappa coefficient *κ* = 0.83, 0.77 and 0.81 shows that there was a good level of concordance between the results of the allelic and genotypic variants evaluated by PCR-RFLP and those detected by the sequencing method of the *IL-1B-511*; *IL-10-1082* and *TNF-A-308* polymorphisms, respectively.

## Discussion

The prevalence of *H. pylori* infection was greater in the low-risk GC population (Tumaco, 94.3%) compared to the high-risk population (Túquerres, 85.65%) [31, 32], but these differences are not significant. However, differences in GC incidence rates exist between these two populations, despite being in close geographic proximity (within 200 km of each other); the incidence is 25 times higher in the Túquerres population, which is located in the Colombian Andes with predominantly Amerindian and European descent, than in the Tumaco population, which is located in the Pacific coast and has a predominantly African ancestry [3, 4, 9]. This phenomenon is similar to the ‘African enigma’, which describes that the *H. pylori* infection is very high in Africa but GC is less frequent [10]; which is similar to what was observed in the Tumaco population, a phenomenon previously reported as the ‘Colombian enigma’ [4]. Accordingly, in the present study and with relation to the severity of gastric lesions, in the high GC population: Túquerres has a higher prevalence of IM (68.2%) than Tumaco (31.8%), which is considered a premalignant histological lesion in the progression to intestinal gastric adenocarcinoma, described in the GC precancerous cascade model [5, 33, 34]. The risk of developing GC is 90 times higher when IM appears compared to individuals with normal mucosa [34]_._ These observations suggest that there are other complementary factors of sociocultural type such as diet and the environment [4, 35], as well as bacterial infection, genetic factors associated to humans and biological factors such a sex and age, which are important for the development and progression of the disease [5, 8, 10, 12, 13, 27, 33, 34].

In this work, we found that men have a three times higher risk of developing ACG than women, which is in agreement with another study that reported a two times higher risk of gastric atrophy in men than in women, and that the disease appears at a younger age in males (<55 years old) than in females [34]. In our study, we observed that the ACG risk increases by five times at ages older than 30 in both studied populations, which is in accordance with previous research carried out with Colombian populations that reported a positive association between atrophic gastritis and IM with patients age (40 and 64 years old) [36]. These findings must motivate people to practice gastroscopy as reported by Haruma *et al* [37], who found that atrophic gastritis and IM significantly increase the GC risk in young Asian patients at ages of 18–29.

According to several reports, genetic polymorphisms that encode for different anti- and pro-inflammatory cytokines are considered a key factor in the process of gastric carcinogenesis. Thus, their study can be used as a tool in the search for variations in the individual genetic susceptibility to GC [5, 8, 12, 15, 16, 27].

We found significant differences in the distribution of frequencies of the *IL-1B-511TT* and *IL-10-1082AA* genotypes between the two populations, being more frequent in the patients from the high GC risk zone (54% and 63.9%, respectively) in comparison to patients from the low risk zone (36.6% and 54.3%, respectively). Similar results were observed by Zabaleta *et al* [43], in which the allelic and genotypic frequencies of *IL-1B-511TT* and *IL10-1082AA* were higher in individuals of high GC risk, Afroamericans (26.8% and 41.2%, respectively) than in Caucasians of low risk (12.1% and 26%, respectively). However, the distribution of frequencies of these genotypes was more elevated in the Colombian populations found in our study. Here, the allelic frequencies of the risk genotypes *IL-1B-511T* and *IL-10-1082A* were higher in patients of GC high risk: Túquerres (72% and 79%, respectively) was higher than in the low risk population of Tumaco (66% and 72%).

The differences in the distribution of the allelic and genotypic frequencies of the *IL-1B-511* and *IL-10-1082* polymorphisms between the two studied populations are influenced possibly by the genetic composition of the ethnic groups from these regions [20, 40, 47–49]. The high frequency of the *IL-1B-511T* high risk allele in the population of Túquerres: high risk population, was similar to the one reported for Latin American populations such as Perú (79%), Venezuela (52%) and Ecuador (62.9%) [24, 38, 44], meanwhile the frequency of the *IL-10-1082A* low risk allele found in the same population was higher and similar to the one reported for Asian and Latin populations [25, 40, 41, 47, 48, 50]. The *IL-1B-511TT* and *IL-10-1082AA* genotypes are associated with a higher risk for GC precursor lesions [24, 45], in Caucasian populations [15, 18], Colombia [25, 40], Venezuela [42], USA [14], Asian and Japan [46]. The previous studies are supported by cumulative evidence showing that *IL-1β* induced by *H. pylori* is a pro-inflammatory cytokine that modulates the biological function of various types of gastric epithelial cells [14], is an *in vivo* inhibitor of gastric acid that initiates and amplifies the inflammatory response and induces hypochlorhydria and gastric atrophy [14, 15, 33]. This cytokine is increased in carriers of the genotypes of the *IL-1B-511*, *IL-1B-31* and IL-1RNA2*A2 genotypes and, in thus, this increases the risk of precancerous lesions and adenocarcinomas of distal type [30, 34, 35]_._

A positive association was found between the *IL-1B-511* polymorphism and ACG in the high risk population. The carrier patients of the *IL-1B-511CT* and *IL-1B-511TT* genotypes displayed a 5.54 and 2.35 higher risk increase of ACG (OR = 5.54; 95% CI, 0.58–52.82); (OR = 2.35; 95% CI, 0.25–21.9). Similar results in Colombian patients have been reported [25], where an association between the *IL-1B-511TT* genotype and IM was described (OR = 4.05; 95% CI, 1.35–12.10). A meta analysis by Peleteiro *et al* [41] also reported a positive association between the *IL-1B-511CT* genotype with IM in a high GC risk population of Caucasian origin, but no association in a population of low GC risk of African origin [37]. The biological effect of the *IL-1β* in the risk of gastric carcionogenesis continues to show disparities as shown in various meta analyses of association [26, 38, 39] and no association [40].

In this study, we found that the *TNF-A-308 AA* genotype was absent in the population with high GC risk, while in the low risk population it is only present in 2.9% of patients from the NAG control group. The allelic and genotypic frequencies for this polymorphism follow the global tendency that exhibits a low frequency of the A mutant allele and a significant increase of the G normal allele [38]. Thus, this polymorphism is not associated with GC precursor lesions in neither of the two studied populations, which is in accordance with the findings reported by Acosta *et al* [38], Torres *et al* [39], Martínez *et al* [40], who worked with Peruvian and Colombian populations. Similar results showing no association were described in the meta analysis of Peleteiro *et al* [41] and in the study by Kato *et al* [42], in Venezuela. As demonstrated by both the literature and this current study, it can be suggested that the *TNF-A-308* polymorphism should not be considered as a predictor for GC risk in Colombian populations. That the *TNF-A-308* polymorphism is not a relevant predictor factor for GC risk in Hispanic populations is a view that is shared by other authors [38].

The *in vivo* function of the pro-inflammatory cytokine *IL-10* is to limit inflammatory responses [16, 17, 29]. The *IL-10-1082GG* genotype is associated with both an elevated production of IL-10 and inhibitory effects over the inflammatory function of other cytokines like *IL-1β* y *TNF-A*. In contrast, the *IL-10-1082AA* genotype is associated with both low *IL-10* concentrations in gastric mucosa and GC [47]. We found that the G allele present in the *IL-10-1082GG+GA* versus *GG* genotypes is significantly associated with ACG in control group patients from the population of Túquerres: high GC risk. This association has not been reported for populations from Tumaco (low GC risk). Previous reports have shown that *IL-10-1082AA* and* IL-10-1082AG* genotypes are associated with GC in Caucasian and Asian populations [13, 26, 27], respectively; the latter having G as the risk allele, which is similar to our observations for high risk populations. We did not identify an association between the *IL-10-1082AA* and the risk of developing GCA, a result that is consistent with previous studies that did not find significant differences in the distribution of *IL-10-1082 AA+GA* versus *GG* genotypes between control and GCA groups (OR = 2.91; 95% CI, 0.76–11.08) [25]. In this respect, our results clearly indicate that individuals from the GC high risk population in Colombia: Túquerres and carriers of the *IL-10-1082*AG mutant genotype have a 5.45 higher risk of developing gastric atrophy in comparison to those carrying the *IL-10-1082AA* genotype (OR = 5.45; 95% CI, 1–30.59) within the same studied population. It is important to mention that this association was not observed in individuals that inhabit populations with low GC risk: Tumaco. Similarly, Loh *et al* [20] has reported that in Asian populations, which are categorised as high GC risk, this genotype is associated with GC (OR = 1.56; 95% CI, 1.23–1.96). Zhou *et al* [50] have reported comparable results (OR = 1.50; 95% CI, 1.06–2.11). This genotype is associated with a low *IL-10* cytokine expression in gastric mucosa, reduction of its anti-inflammatory effect and subsequent loss of inhibition of the pro-inflammatory effect provided by cytokines such as *IL-β* and *TNF-A* [47] and induced by *H. pylori* infection.

Several authors have proposed that the variabilities in the association of the polymorphisms with precancerous lesions and GC in populations around the world are the consequences of differences in the genetic composition of ethnic groups, which affect their frequencies [9, 10, 40, 41, 43, 48]. Our results are in accordance with this idea since the distribution of the genotypic and allelic risk polymorphisms are significantly different between the two studied populations, which displays contrasting features with respect to the ethnic origin (being mostly African in Tumaco and mostly Amerindian in Túquerres). Also in our study, we show strong evidence supporting an association between the *IL-10-1082AG* mutant phenotype and ACG. It is possible that the previously described evidence in combination with the coevolutionary theory demonstrated in our study (disruption of the host-*H. pylori* coevolution associated with more severe gastric lesions) [9] determine biological relationships that are less favorable for the host and explain partially the differences between the risk and incidence of GC between the two Colombian populations analysed in this work.

A limitation of this study is the relatively small sample size of some subgroups, particularly the Tumaco population and the patients with gastric atrophy. Although statistically significant associations were detected, a larger sample size would increase statistical power and might reveal additional genotype–phenotype relationships.

The identification of *IL-10-1082AG* as a genetic risk factor for gastric atrophy in high-risk Colombian populations suggests potential clinical applications. This polymorphism could be incorporated into future risk-stratification models to identify individuals who may benefit from closer endoscopic surveillance or early *H. pylori* eradication. In high-incidence regions such as Túquerres, genetic screening combined with histological and microbial markers could improve targeted prevention strategies and reduce progression toward GC.

## Conclusion

In conclusion, the disparity of the GC risk in the two populations can be the result of (i) the significant differences in the distribution of the allelic and genotypic differences of *IL-1B-511* and *IL-10-1082* polymorphisms and (ii) the positive association between the *IL-10-1082AG* genotype with ACG in the high GC risk population in Colombia. Nevertheless, future studies that include the genetic ancestry and the disruption of the *H. pylori*-host coevolution are needed to improve the understanding of the role of the cytokine polymorphisms in the gastric carcinogenesis.

## Conflicts of interest

The authors declare that they have no competing interests.

## Funding

The Molecular Microbiology Lab and Public Health Research Group from Universidad de Nariño supported this investigation. The Universidad del Valle and Universidad Cooperativa de Colombia provided financial support for the co-execution of this research project. Our RC No 693-2014 research grant was financed by the National Program CTel in Health of MINCIENCIAS-Colombia.

## Author contributions

All the authors that were involved in the acquisition and interpretation of the results, read and approved the final manuscript; Guzman K, Pazos AJ, Bravo LE and Pabon V designed the research; Pazos A, Pabon V, Rosero C and Angulo A conducted the microbiological and molecular tests; Pazos A, Pabon V, Angulo A, Guzman K and Hidalgo A, analysed the data; Pazos A, Pabon V, Hidalgo A, Angulo A, Rosero C, Guzman K and Bravo LE wrote, edited and revised the manuscript.

## Figures and Tables

**Table 1.** Distribution of the association of sociodemographic characteristics and H. pylori infection with the type of gastric lesion.

Characteristic		ACG	NAG	OR	IC (95%)		*p* value
		*n* (%)	*n* (%)		LI	LS	
Gender							0.001
	Female	21 (12.8)	143 (87.2)	1.00			
	Male	31 (30.7)	70 (69.3)	3.02	1.62	5.63	
Age (years)							0.011
	≤30	2 (5.0)	38 (95.0)	1.00			
	˃30	50 (22.2)	175 (77.8)	5.43	1.27	23.29	
*H. pylori* infection							0.732
	Negative	5 (17.2)	24 (82.8)	1.00			
	Positive	47 (19.9)	189 (80.1)	1.19	0.43	3.29	

**ACG** = atrophic chronic gastritis; **NAG** = non-atrophic gastritis

**Table 2.** Genotypic and allelic frequencies of cytokine polymorphisms according to GC risk population.

Genotypes Alleles	Population					
	Túquerres		Tumaco		*p* value	
	High GC risk		Low GC risk			
*IL-1B-511*	*n*	%	*n*	**%**	0.006	
CC	15	9.50	7	6.90		
CT	57	36.1	57	56.4		
TT	86	54.4	37	36.6		
Alleles						
C	88	0.28	69	0.34		
T	228	0.72	133	0.66		
*TNF-A-308*					0.009	
GG	143	89.9	82	78.1		
GA	16	10.1	20	19.0		
AA	-	-	3	2.90		
Alleles						
G	302	0.95	177	0.85		
A	16	0.05	33	0.15		
*IL-10-1082*					0.011	
GG	10	6.30	19	18.1		
GA	47	29.7	29	27.6		
AA	101	63.9	57	54.3		
Alleles						
G	64	0.21	67	0.28		
A	252	0.79	143	0.72		

**Table 3.** Genotypic frequencies of cytokine polymorphisms according to the lesion and GC risk population.

Genotypes	GC high risk					GC low risk				
	Lesion type				*p* value	Lesion type				*p* value
	NAG		ACG			NAG		ACG		
*IL-1B-511*	*n*	%	*n*	%	0.09	*n*	%	*n*	%	0.28
CC	14	11.0	1	3.20		4	4.94	3	15.0	
CT	41	32.3	16	51.6		47	58.0	10	50.0	
TT	72	56.7	14	45.2		30	37.0	7	35.0	
CT + TT versus CC	113	89.0	30	96.8	0.18	77	95.1	17	85.0	0.11
*TNF-A-308*					0.60					0.12
GG	115	90.6	28	87.5		63	74.1	19	95.0	
GA	12	9.40	4	12.5		19	22.4	1	5.00	
AA	-	-	-	-		3	3.50	-	-	
GA + AA versus CC	12	9.40	4	12.5	0.60	22	25.9	1	5.00	0.04
*IL-10-1082*					0.19					0.62
GG	6	4.80	4	12.5		15	17.60	4	20.0	
GA	40	31.7	7	21.9		22	25.90	7	35.0	
AA	80	63.5	21	65.6		48	56.50	9	45.0	
GA + AA versus GG	120	95.2	28	87.5	0.10	70	82.40	16	80.0	0.28

**NAG**: Non-atrophic gastritis; **ACG**: Atrophic chronic gastritis

**Table 4.** Risk of developing gastric atrophy according to cytokine genotypes in populations with contrast in the risk of GC.

Genotypes	ACG High risk				ACG Low risk				
	OR	CI		*p* value	OR	CI		*p* value	
*IL-1β-511*									
CC	1.00				1.00				
CT	5.54	0.58	52.82	0.13	0.90	0.55	43.31	0.15	
TT	2.35	0.25	21.90	0.35	0.80	0.49	6.53	0.37	
*IL-10-1082*									
AA	1.00				1.00				
AG	5.45	1.00	30.59	**0.05**	2.80	0.50	15.78	0.24	
GG	0.67	0.23	1.97	0.47	0.15	0.31	4.22	0.83	

ACG = atrophic chronic gastritis; CI = confidence interval; OR = odds ratio simultaneously adjusted by sex, age, *H. pylori* infection and by all cytokine polymorphisms
